# Differentiation of Cerebral Dissecting Aneurysm from Hemorrhagic Saccular Aneurysm by Machine-Learning Based on Vessel Wall MRI: A Multicenter Study

**DOI:** 10.3390/jcm11133623

**Published:** 2022-06-23

**Authors:** Xin Cao, Yanwei Zeng, Junying Wang, Yunxi Cao, Yifan Wu, Wei Xia

**Affiliations:** 1 Department of Radiology, Huashan Hospital, Fudan University, Shanghai 200040, China; 13262566515@163.com (X.C.); zywradiol@163.com (Y.Z.); 17896381815@163.com (Y.W.); 2 Greater Bay Area Institute of Precision Medicine (Guangzhou), Guangzhou 511466, China; 3 Department of Medical Imaging, The First Affiliated Hospital of Shandong First Medical University & Shandong Province Qianfoshan Hospital, Jinan 250014, China; jywang1120@163.com; 4 Radiology Academy, Shandong First Medical University & Shandong Academy of Medical Sciences, Taian 271016, China; caopangxin@163.com; 5 Suzhou Institute of Biomedical Engineering and Technology, Chinese Academy of Sciences, Suzhou 215163, China

**Keywords:** aneurysm, vessel wall magnetic resonance imaging, radiomics, machine-learning, external verification

## Abstract

The differential diagnosis of a cerebral dissecting aneurysm (DA) and a hemorrhagic saccular aneurysm (SA) often depends on the intraoperative findings; thus, improved non-invasive imaging diagnosis before surgery is essential to distinguish between these two aneurysms, in order to provide the correct formulation of surgical procedure. We aimed to build a radiomic model based on high-resolution vessel wall magnetic resonance imaging (VW-MRI) and a machine-learning algorithm. In total, 851 radiomic features from 146 cases were analyzed retrospectively, and the ElasticNet algorithm was used to establish the radiomic model in a training set of 77 cases. A clinico-radiological model using clinical features and MRI features was also built. Then an integrated model was built by combining the radiomic model and clinico-radiological model. The area under the ROC curve (AUC) was used to quantify the performance of models. The models were evaluated using leave-one-out cross-validation in a training set, and further validated in an external test set of 69 cases. The diagnostic performance of experienced radiologists was also assessed for comparison. Eight features were used to establish the radiomic model, and the radiomic model performs better (AUC = 0.831) than the clinico-radiological model (AUC = 0.717), integrated model (AUC = 0.813), and even experienced radiologists (AUC = 0.801). Therefore, a radiomic model based on VW-MRI can reliably be used to distinguish DA and hemorrhagic SA, and, thus, be widely applied in clinical practice.

## 1. Introduction

A cerebral dissecting aneurysm (DA), as a clinical emergency, has different a pathophysiological mechanism and etiology from a common cerebral saccular aneurysm (SA) [1]. Under arterial pressure, the blood enters the arterial wall from the tear of intima to form intramural hematomas (IMH), which are usually located in the media layer [2]. A prior study shows that the annual rupture rate of DA is nearly 58%, increasing the risk of subarachnoid hemorrhage and ischemic stroke [3,4], while the annual rupture rate of SA is 0.95%. DA has a worse clinical outcome, with higher risk of rupture with than SA [5,6,7]. A diameter greater than 10 mm is an independent risk factor for re-bleeding of an aneurysm [8]. The preferred treatment options of DA are vessel occlusion, surgical trapping, or flow diverter [9], whereas clipping or stenting is more frequently used for SA. Some hemorrhagic SAs with hematoma and thrombus are difficult to differentiate from DA.

Vessel wall magnetic resonance imaging (VW-MRI) provides non-invasive, reliable measurements for the visualization of the vessel wall, lumen, and intimal tear [10,11], which is recommended as an optimal diagnostic technique for DA [12,13,14]. IMH usually shows high signal intensity in T1-weighted imaging (T_1_WI). Hemorrhagic SA with mural thrombus or blood swirls sometimes has similar MRI signs to DA. Thus, constructing a diagnostic model through machine-learning methods, which fully utilizes the 3D T_1_WI of VW-MRI data, could be very helpful. Radiomics has gradually been applied in the field of aneurysms. Podgorsak et al. study a convolutional neural network that automatically extracts blood flow-related radiomic features of intracranial aneurysms [15]. Some applications of artificial intelligence techniques for aneurysm detection, risk stratification, and prognosis prediction are reported [16,17]. However, the use of radiomic features to identify the classification of aneurysm has not been studied before. Therefore, this research aimed to build a VW-MRI-based radiomic model for differentiating DA from hemorrhagic SA, and to validate its generalizability.

## 2. Methods

This retrospective study was approved by the Institutional Review Board of Huashan Hospital, affiliated with Fudan University, and the First Affiliated Hospital of Shandong First Medical University, and the need for informed consent was waived by the ethical committees of both hospitals. We collected medical data retrospectively from January 2017 to January 2022 in both hospitals.

## 3. Participants

A total of 255 patients who underwent a high-resolution (HR) VW-MRI and had an aneurysm of the artery in the brain or neck detected were recruited. The subjects were included based on the following criteria: (i) final diagnosis based on findings during surgery; (ii) completed the imaging examination within 72 h after admission; (iii) large aneurysm (long diameter ≥ 7 mm) with mixed MRI signals; and iv) the MRI images show details with good or excellent quality. The exclusion criteria were as follows: (i) past neurosurgery history or any other neurological diseases, such as cerebral hemorrhage, brain tumors, ischemic stroke, brain trauma, arteriovenous malformations, etc.; (ii) poor image quality due to motion artifact; and (iii) small aneurysms with homogeneous low signal on MRI. Clinical information was recorded, including age, gender, clinical symptoms, lesion size, and lesion location. The international normalized ratio (INR) was recorded as an indicator of the patient′s coagulation status. Personal information was desensitized prior to analysis. To facilitate subsequent analysis, DA patients were defined as negative samples, and SA patients were marked as positive samples.

### 3.1. Image Data Acquisition

All MRIs were performed using two 3.0-T magnetic resonance systems of the same model in two hospitals (Discovery MR 750, GE Healthcare, Milwaukee, MI, USA). The patients in our hospital, as training sets, receive scanning parameters as follows: (i) 3D T_1_WI: repetition time (TR) = 600 ms, echo time (TE) = 14.8 ms, field of view (FOV) = 240 × 240 mm^2^, matrix = 256 × 256, signal noise ratio (SNR) = 1.00, slice thickness = 1.0 mm, and scanning time = 4′15′′; (ii) time-of-flight (TOF) magnetic resonance angiography (MRA): TR = 23 ms, TE = 3.5 ms, FOV = 160 × 160 mm^3^, flip angle = 20°, and scanning time = 5′20″. The patients in another hospital were treated as validation sets, and the scanning parameters were as follows: (i) 3D T_1_WI: TR = 600 ms, TE = 14.4 ms, FOV = 200 × 200 mm^2^, matrix = 288 × 288, SNR = 1.00, slice thickness = 1.0 mm, and scanning time = 4′30; (ii) TOF-MRA: TR = 25 ms, TE = 3.4 ms, FOV = 220 × 220 mm^3^, flip angle = 20°, and scanning time = 3′48″.

### 3.2. Image Processing and Radiomic Feature Extraction

ITK-SNAP software v.3.8.0 (the University of Pennsylvania and the University of Utah, open source, http://www.itksnap.org/pmwiki/pmwiki.php (accessed on 1 January 2022) was used for 3D manual segmentation. Region of interest (ROI) was drawn on all 3D T_1_WI, slice-by-slice, on the original sagittal images, reconstructed axial images, and reconstructed coronal images. The maximum range of ROI was drawn along the visible border to cover the entire volume of aneurysm. Reader 1, with 7 years of experience in neurovascular imaging, drew the ROI delineation twice within the first week, following the same procedure. In addition, Reader 2, with more than 11 years of experience, drew an ROI separately, to assess the consistency between observers by comparing with the results of Reader 1. To synchronize the acquired images, the mean value and standard deviation (SD) of the image intensity were calculated, and images were standardized by z-score method [18]. Radiomic features were extracted using the Pyradiomics v.2.2.0 in Python [19]. To further investigate the heterogeneity within ROI, wavelet filters were applied to the original images, transforming the original images into versions focused on information at different scales [19]. Details of these features are provided in the documentation for PyRadiomics (https://pyradiomics.readthedocs.io/en/2.2.0/index.html (accessed on 1 January 2022)).

### 3.3. Model Building

The intraclass correlation coefficients (ICC) of each feature were calculated; the feature with ICC < 0.75 was considered unreliable and discarded. Reader 1 accounted for the samples if good agreement (ICC > 0.75) was achieved. Spearman correlation analysis was performed to obtain the mutual correlation between features. Two features were considered highly correlated if the pairwise correlation coefficient reached 0.9, and the feature with the largest mean absolute correlation coefficient was redundant and eliminated.

To avoid the risk of overfitting, the ElasticNet [20] was used for model building. Due to the limited number of cases in the training set, too many features in the model may increase the probability of overfitting. The ratio of L1 and L2 penalties in ElasticNet were set to 1 and 0, respectively, to select the most relevant features, and reduce the feature number. The ElasticNet was trained using 5-fold cross-validation to select radiomic features and determine the corresponding weights, then a radiomic model was constructed as the weighted sum of the selected radiomic features. In addition, a clinico-radiological model was established using clinical features and MRI features. The features in the clinico-radiological model were determined by multivariate logistic regression with backward feature selection, and the Akaike information criterion was used as the quantitative indicator for feature selection. Finally, an integrated model, which incorporated the radiomic model and the clinico-radiological model, was built by multivariate logistic regression.

### 3.4. Model Evaluation and Statistical Analysis

Models were evaluated by the leave-one-out cross-validation (LOOCV) method in the training set [21]. The diagnostic performance of the models was quantified by the area under the receiver operating characteristic (ROC) curve (AUC). The accuracy (ACC), sensitivity (SEN), and specificity (SPE) of each model were calculated. The Mann–Whitney U test and chi-square test were used to compare the differences between DA and hemorrhagic SA, respectively. All statistical tests were two-sided; *p* < 0.05 was considered statistically significant.

### 3.5. Radiologists′ Diagnosis

Each diagnosis was made by three junior neuroradiologists, and confirmed by two more senior neuroradiologists with more than 10 years’ working experience, who were blind to clinical information and surgical outcomes. The diagnosis of an SA was based on the shape and the flowing void effect low signal in the aneurysm. Hemorrhagic SAs contain hematoma, thrombus, or intra-aneurysmal flow artifacts, which show high signal or mixed signal on T_1_WI. The diagnosis of a DA requires some specific MRI features, such as IMH, double lumen, or the intimal flap. When the diagnosis diverged, the opinion of two senior neuroradiologists was decisive.

Model building, evaluation, and statistical analysis using R programming language (version 3.4.3, R Core Team, Vienna, Austria).

## 4. Results

**Figure 1:**
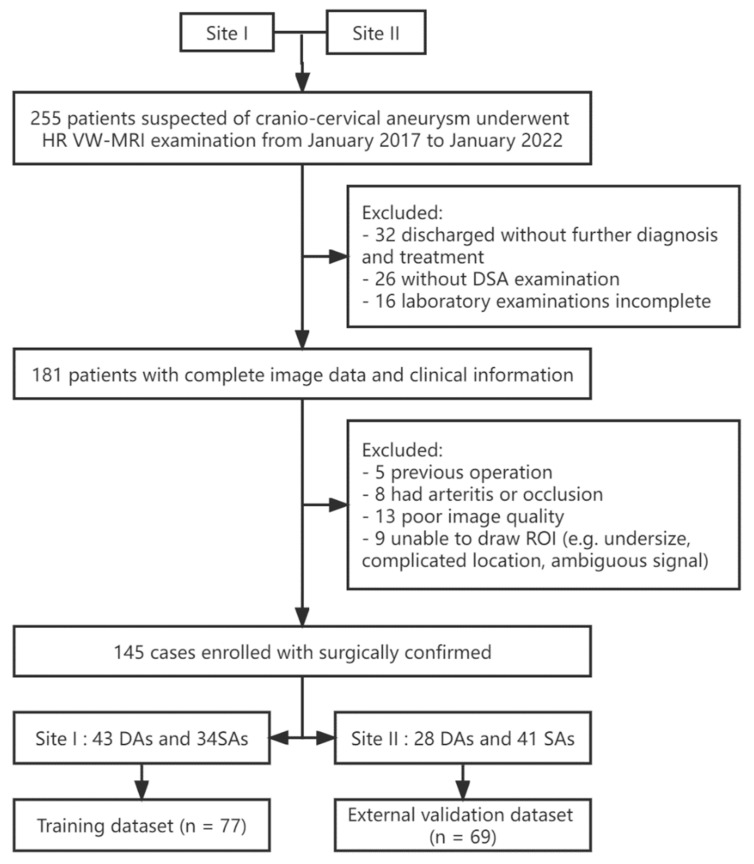
Flow chart of patient recruitment, inclusion, and exclusion criteria for the dataset.

Of the 145 patients included in this study, 77 cases from our hospital constitute the training set, and 69 cases from another hospital constitute the external test set (Figure 1).

No significant differences are found in the age, symptoms, lesion size, INR, or sign resembling the intimal flap of MRI in the training set, and no differences are found in INR in the external test set (Table 1).

The formula of the clinico-radiological model:

(1) $$\begin{array}{ll} \mathrm{Clinico} & -\mathrm{radiological}\;\mathrm{score}\\ & =2.737+19.737\times \mathrm{Sign}.\mathrm{intimal}\;\mathrm{flap}-20.268\times \mathrm{HHT}\;\\ & -0.901\times \mathrm{Long}.\mathrm{diameter}-2.574\times \mathrm{location} \end{array}$$

**Figure 2:**
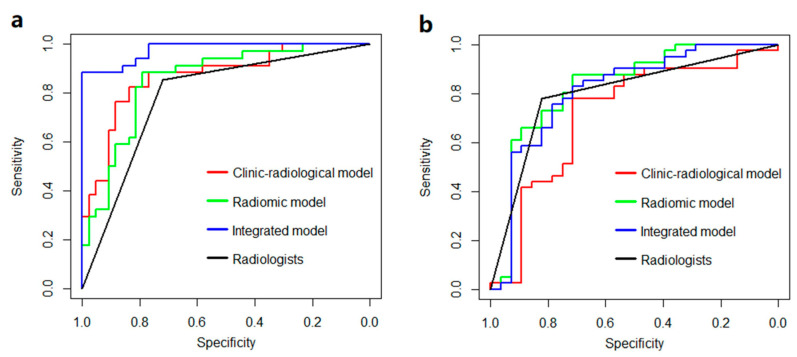
The ROC curve of models and radiologists. (**a**) training set, (**b**) external test set.

The ROC curve of the clinico-radiological model is illustrated in Figure 2a.

The diagnostic performance of the clinico-radiological model is presented in Table 2.

In the radiomic model, eight radiomic features are included: one shape feature (elongation), which shows the relationship between the two largest principal components in the ROI shape, and its value ranges from 0 (line-like object) to 1 (circle-like object). The remaining seven features are wavelet features. These wavelet features quantify the inhomogeneity of aneurysm intensity from different aspects. The formula of the radiomic model:

(2) $$\begin{array}{ll} \mathrm{Radiomic}\mathrm{score} & =13.490+0.628\times \mathrm{shape}.\mathrm{Elongation}+1.980\times \mathrm{LLH}.\mathrm{firstorder}.\mathrm{Mean}\\ & -2.610\times \mathrm{LHL}.\mathrm{gldm}.\mathrm{DependenceEntropy}-0.012\times \mathrm{LHH}.\mathrm{firstorder}.\mathrm{Skewness}\\ & +0.002\times \mathrm{HLL}.\mathrm{gldm}.\mathrm{LargeDependenceEmphasis}-116.438\times \mathrm{HHL}.\mathrm{firstorder}.\mathrm{Mean}\\ & -8.674\times \mathrm{HHH}.\mathrm{glcm}.\mathrm{Imc}2+0.552\times \mathrm{LLL}.\mathrm{glcm}.\mathrm{ClusterShade} \end{array}$$

**Figure 3:**
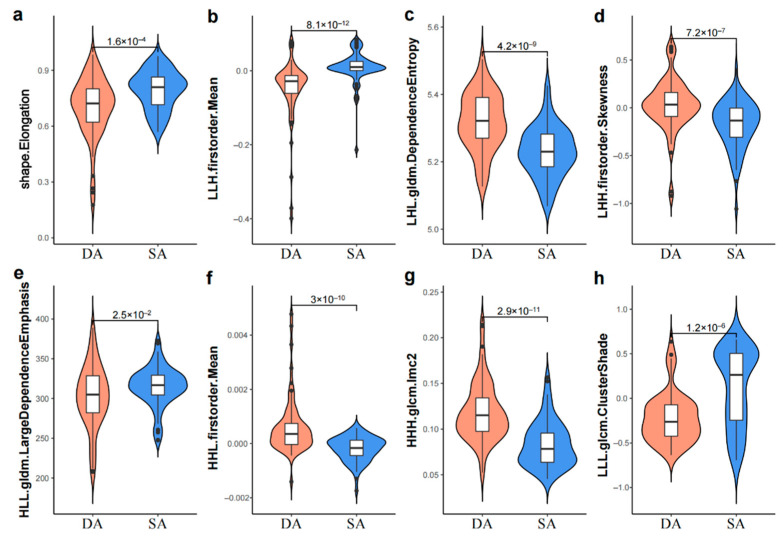
The violin plots of eight features (**a**–**h**) in the radiomic model.

The radiomic score is transformed to the probability of DA or hemorrhagic SA by the sigmoid function in Equation (2). The extracted radiomic features are used as input, and the radiomic model yields the probability for each patient. The violin plots of the features in the ElasticNet-based radiomic model are illustrated, to show how different features are distributed in patients (Figure 3). 

**Figure 4:**
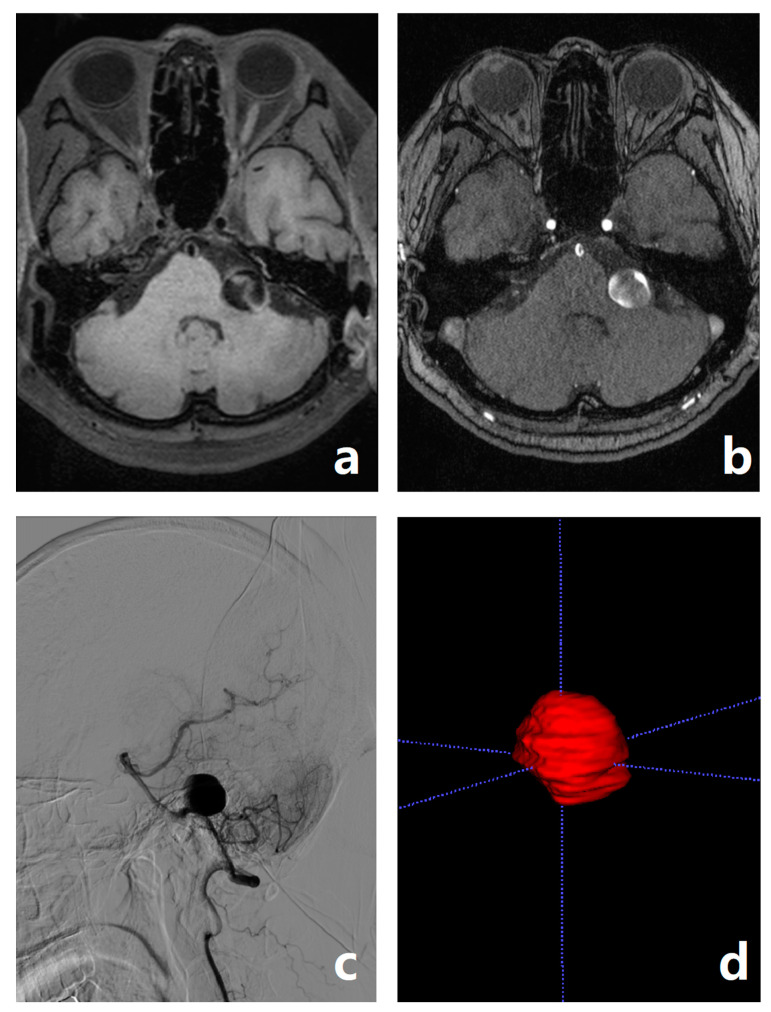
A 64 year old female who had headache and nausea for two weeks. The radiologist diagnosed a DA in the V4 segment of her left vertebral artery. There are double lumen and sign resembling the intimal flap in the aneurysm on 3D T_1_WI (**a**). TOF-MRA shows inhomogeneous signal within the aneurysm (**b**). DSA finds no bleeding site or intimal tear hole (**c**). Her 3D volumetric reconstruction of the ROI (**d**). The radiomic model diagnoses it as an SA, which is consistent with the surgical results.

Figure 4 shows an example of representative MRI images.

In the training set, the integrated model improves the diagnostic performance in terms of AUC, ACC, and SPE (Table 2), which is superior to both the radiomic model and the clinico-radiological model (Figure 2a). When applying these models to the external validation set, the radiomic model performs the best, even better than the experienced radiologists (Figure 2b). 

## 5. Discussion

HR VW-MRI provides useful information to distinguish different types of aneurysms [14,22,23], and this is the first time using the machine-learning method of multicenter external verification to distinguish DA from hemorrhagic SA [24]. Unlike several previous studies, that only draw ROI from a single image slice [25], the ROIs in this study are drawn in all slices with a thickness of only 1.0 mm, which contains sufficient data information. ElasticNet, as a compression estimation method for variable selection, is suitable for the selection of biomarkers in high-dimensional data [20,26,27]. In addition to surpassing the method of selecting predictors based on the strength of their univariable association with the outcome, it also enables the panel of selected features to be combined into a radiomic signature [20,28,29].

Eight potential predictors were selected from 851 candidate radiomic features for radiomic model construction. For the shape feature (elongation), our result is consistent with some studies in which the proportion of non-saccular morphology is higher in posterior circulation aneurysms than in anterior circulation aneurysms [5,30]. Shape-based features suggest a high correlation between aneurysm type and morphological properties. This phenomenon can perhaps be explained by vascular morphology and hemodynamics. The arteries in the posterior circulation are relatively straight, and the blood flow enters the middle layer through the intimal tear hole to form a dissection [31], making the long diameter of DA larger than the short diameter. The remaining seven features are wavelet features which quantify the heterogeneity of aneurysm MRI intensity from different aspects, indicating that wavelet features are more discriminative [32,33], and provide more supplementary information to the shape-based features [34]. Some of the wavelet features are mentioned in previous studies, which are mainly related to the heterogeneity of tumors, and used for tumor grading and prognosis evaluation [35,36,37,38,39]. We infer that they may have varying degrees of correlation with the mixed signals of hemorrhage or the intimal flap. The extraction and calculation of radiological features are based on mathematical formulas of machine vision, and it is difficult to explain the relationship between higher-order features and pathological manifestations [40].

The AUC of both the clinico-radiological model and integrated model in the training set are much higher than the external test set, which is most likely a hint of overfitting. Overfitting is the phenomenon of matching a particular dataset too closely or precisely to fit other data well, which shows that the generalization of these two models is limited. The diagnostic performance of the radiomic model is similar in both sets, which demonstrates that it has good repeatability and generalization. The radiomic model performs better than the other two models in the test set, which proves that it has good diagnostic efficiency and application value. In addition, the radiomic model exceeds that of experienced radiologists in this study. This means that the application of machine-learning in clinical work improves the accuracy of differential diagnosis, reduces the probability of misdiagnosis, reduces the workload of radiologists, and helps surgeons to choose the most suitable surgical plan. We believe that the problem of insufficient generalization ability of radiomics models based on MRI can be solved by further algorithm optimization of image data and processing.

There are still several limitations. First, the sample size was not large enough; however, we used LOOCV in the training set, and there was an external test set to evaluate the performance of the models. Second, the time-consuming and labor-intensive process of manual segmentation may cause bias; automatic segmentation should be applied in future radiomics studies. Third, only a 3D T_1_WI sequence was used, while models based on multimodal MRI may perform better. Fourth, in future research, we will unify the image acquisition protocols in multicenters in prospective studies to ensure the homogeneity of the image.

In conclusion, via multicenter external validation, the presented radiomic model is an effective tool for preoperative differentiation of DA from a hemorrhagic SA, with good accuracy and generalizability, which can provide supplementary diagnoses to radiologists, and assist the surgeon to make the most suitable operation plan.

## Figures and Tables

**Table 1:** Clinical characteristics and MRI features of patients enrolled.

	Training Set (*n* = 77)			External Test Set (*n* = 69)		
	DA	SA	* p *	DA	SA	* p *
** No. of patients **	43	34		28	41	
** Female (*n*, %) **	13 (30.23%)	23 (67.65%)	0.001	9 (32.14%)	32 (78.05%)	<0.001
** Age (year) **	49.79 ± 12.06	55.00 ± 13.55	0.094	54.29 ± 11.43	57.54 ± 14.91	0.035
** Clinical symptoms (*n*, %) **	37 (86.05%)	24 (70.59%)	0.097	11 (39.29%)	32 (78.05%)	0.001
** MRI features (*n*, %) **						
Sign resembling the intimal flap	21 (48.84%)	23 (67.65%)	0.098	10 (35.71%)	26 (63.42%)	0.024
HHT	34 (79.07%)	12 (35.29%)	<0.001	19 (67.86%)	14 (34.15%)	0.006
** Size (cm) **						
Long diameter	1.91 ± 1.04	1.66 ± 0.92	0.543	1.47 ± 0.80	1.94 ± 0.95	0.004
Short diameter	1.15 ± 0.60	1.30 ± 0.73	0.203	1.22 ± 0.75	1.59 ± 0.78	0.005
** Lesion location (*n*, %) **						
** Anterior circulation **	13 (30.23%)	26 (76.47)	<0.001	5 (17.86%)	33 (80.49%)	<0.001
ICA	11 (25.58%)	18 (52.94%)	0.014	1 (3.57%)	23 (56.10%)	<0.001
MCA	2 (4.65%)	9 (26.47%)	0.007	4 (14.29%)	9 (21.95%)	0.424
** Posterior circulation **	30 (69.77%)	7 (20.59%)	<0.001	23 (82.14%)	8 (19.51%)	<0.001
BA	4 (9.30%)	2 (5.88%)	0.578	5 (14.86%)	1 (2.44%)	0.026
VA	24 (55.81%)	2 (5.88%)	<0.001	18 (64.29%)	3 (7.32%)	<0.001
PCA	2 (4.65%)	3 (8.82%)	0.461	0	4 (9.76%)	0.089
** Coagulation examination **						
INR	0.97 ± 0.06	0.96 ± 0.10	0.372	0.96 ± 0.05	0.96 ± 0.13	0.214

MRI, magnetic resonance imaging; HHT, hemorrhage, hematoma, or thrombus; ICA, internal carotid artery; MCA, middle cerebral artery; BA, basilar artery; VA, vertebral artery; PCA, posterior cerebral artery; INR, international normalized ratio.

**Table 2:** Diagnostic performance of models and radiologists.

Model or Radiologists	Training Set				External Test Set			
	AUC	ACC	SEN	SPE	AUC	ACC	SEN	SPE
Clinico-radiological model	0.867	0.831	0.823	0.837	0.717	0.753	0.780	0.714
Radiomic model	0.853	0.831	0.882	0.791	0.831	0.812	0.878	0.714
Integrated model	0.977	0.948	0.882	1.000	0.813	0.782	0.829	0.714
Radiologists	0.787	0.779	0.852	0.720	0.801	0.797	0.780	0.821

AUC, area under the ROC curve; ACC, accuracy; SEN, sensitivity; SPE, specificity. The dissecting aneurysm is defined as negative and the saccular aneurysm is defined as positive.

## Data Availability

The data presented in this study are available on request from the corresponding author. The data are not publicly available due to protecting patient privacy.
